# Structural basis of dynamic P5CS filaments

**DOI:** 10.7554/eLife.76107

**Published:** 2022-03-14

**Authors:** Jiale Zhong, Chen-Jun Guo, Xian Zhou, Chia-Chun Chang, Boqi Yin, Tianyi Zhang, Huan-Huan Hu, Guang-Ming Lu, Ji-Long Liu

**Affiliations:** 1 School of Life Science and Technology, ShanghaiTech University Shanghai China; University of Virginia United States; National Heart, Lung and Blood Institute, National Institutes of Health United States

**Keywords:** P5CS, cytoophidium, proline synthesis, *Drosophila*, Cryo-EM, metabolic enzyme, *D. melanogaster*

## Abstract

The bifunctional enzyme Δ^1^-pyrroline-5-carboxylate synthase (P5CS) is vital to the synthesis of proline and ornithine, playing an essential role in human health and agriculture. Pathogenic mutations in the P5CS gene (ALDH18A1) lead to neurocutaneous syndrome and skin relaxation connective tissue disease in humans, and P5CS deficiency seriously damages the ability to resist adversity in plants. We have recently found that P5CS forms cytoophidia in vivo and filaments in vitro. However, it is difficult to appreciate the function of P5CS filamentation without precise structures. Using cryo-electron microscopy, here we solve the structures of *Drosophila* full-length P5CS in three states at resolution from 3.1 to 4.3 Å. We observe distinct ligand-binding states and conformational changes for the GK and GPR domains, respectively. Divergent helical filaments are assembled by P5CS tetramers and stabilized by multiple interfaces. Point mutations disturbing those interfaces prevent P5CS filamentation and greatly reduce the enzymatic activity. Our findings reveal that filamentation is crucial for the coordination between the GK and GPR domains, providing a structural basis for the catalytic function of P5CS filaments.

## Introduction

The bifunctional enzyme Δ^1^-pyrroline-5-carboxylate synthase (P5CS) is responsible for proline and ornithine metabolism (Baumgartner et al., 2000; Baumgartner et al., 2005; Hu et al., 2008; Pérez-Arellano et al., 2010). In humans, over 30 mutations in P5CS have been identified as the causes of rare diseases (Baumgartner et al., 2000; Baumgartner et al., 2005; Marco-Marín et al., 2020; Pérez-Arellano et al., 2010; Skidmore et al., 2011). In addition, the glutamine-proline regulatory axis has been considered a promising target for cancer therapy (Guo et al., 2020; Liu et al., 2012). In plants, proline synthesis is associated with plant stress resistance (Pérez-Arellano et al., 2010). Therefore, P5CS is of great significance in human health and agriculture.

Previous studies have revealed a characteristic compartmentation of enzymes via filamentation (Hunkeler et al., 2018; Johnson and Kollman, 2020; Liu, 2010; Park and Horton, 2019; Stoddard et al., 2020). This filamentous structure is membraneless and termed the cytoophidium for its appearance (Liu, 2010; Liu, 2016). The cytoophidium has emerged as a mechanism for the regulation of metabolic enzymes (Hansen et al., 2021; Liu, 2016; Zhou et al., 2021). Recently, we have shown that *Drosophila* P5CS forms cytoophidia in vivo and forms individual filaments in vitro (Zhang et al., 2020).

P5CS corresponds to two individual proteins in prokaryotes and some lower eukaryotes such as yeast. One is the glutamate kinase (GK, *pro*B gene), and the other is γ-glutamyl phosphate reductase (GPR, *pro*A gene) in *Escherichia coli*. Kinetic analysis suggest that bacterial GK and GPR form a complex (Gamper and Moses, 1974). The dual functions of P5CS in higher eukaryotes implicate that both GK and GPR have evolved into one single protein for coupling reactions. However, no structure of the full-length P5CS has been solved. The underlying mechanisms of the catalytic reaction and the function of filamentation remain unknown.

Using cryo-electron microscopy (cryo-EM), here we solve the structures of full-length P5CS in multiple filamentous states. We reconstruct *Drosophila* P5CS structures at 3.1–4.3 Å resolutions, providing detailed information of the P5CS filaments bound with different ligands. Our results describe the assembly mechanism of P5CS filaments, in which the GK domain forms tetramer and the GPR domain forms dimer structure, and both domains form specific interaction interfaces. Based on these structures, we propose a working model that filamentation is critical for the coordinated reactions between GK and GPR, the two domains of P5CS.

## Results

### Overall structures of P5CS filaments

**Figure 1.**
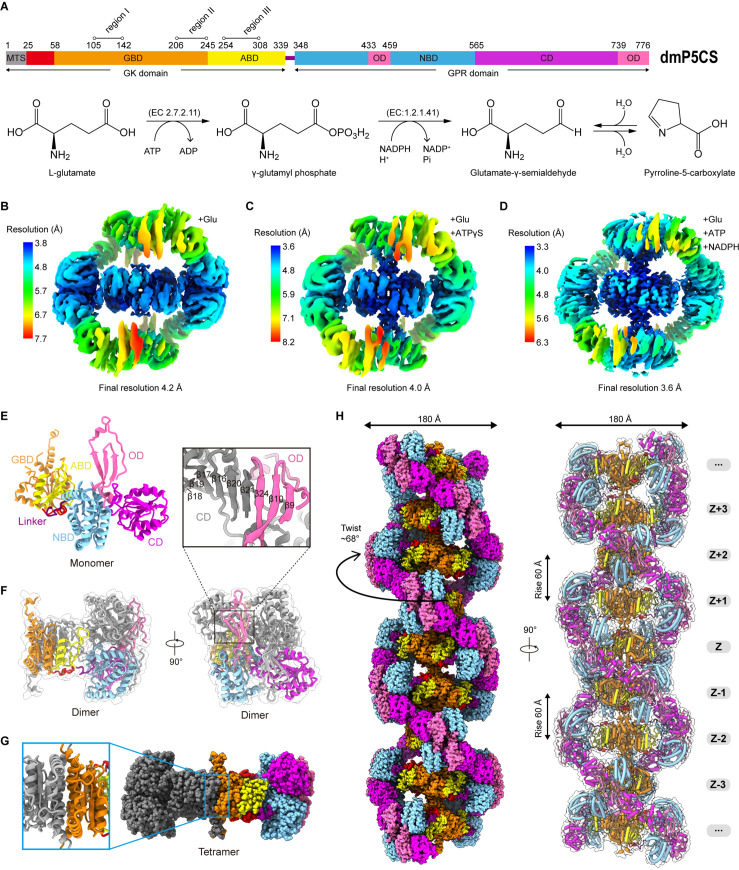
Bifunctional enzyme properties and cryo-electron microscopy (cryo-EM) analysis of P5CS filaments. (**A**) Domain organization of *Drosophila melanogaster* P5CS, which consists of two domains, N-terminal glutamate kinase (GK) domain and C-terminal γ-glutamyl phosphate reductase (GPR) domain. Putative mitochondrial targeting sequence (MTS) is labeled in gray; the glutamate-binding domain (GBD) and the ATP-binding domain (ABD) of the GK domain are respectively shown in orange and yellow; the NADPH-binding domain (NBD), the catalytic domain (CD), and the oligomerization domain (OD) of the GPR domain are shown in cyan, purple, and pink, respectively. Bifunctional P5CS enzyme catalytic reaction and residue numbers for domain boundaries are shown. (**B–D**) Single-particle analysis for 3D reconstruction of P5CS filaments, three cryo-EM maps of P5CS^Glu^ filament, P5CS^Glu/ATPγS^ filament, and P5CS^Mix^ filament are colored by local resolution estimations. (**E**) The structures of the P5CS monomer and color codes for P5CS models are indicated. (**F**) The P5CS dimer. Two monomers (gray or color coded by domain) interact via GPR domain hairpins contact. (**G**) The P5CS tetramer (sphere representation) is formed via GK domain interaction (cartoon representation) between two P5CS dimers (gray or color coded by domain). (**H**) The sphere and cartoon representation of P5CS filaments. P5CS filaments are modeled by the cryo-EM map. The rotated view is shown in the right panel; its rise, twist, and width are indicated.

The P5CS molecule contains two domains, GK and GPR, catalyzing the first and second steps in the biosynthesis of proline from glutamate. The GK domain catalyzes glutamate phosphorylation, and the GPR domain catalyzes the NADPH-dependent reduction of γ-glutamyl phosphate (G5P) to glutamate-γ-semialdehyde (GSA). The end product P5C, formed by a spontaneous cyclization reaction of GSA (Figure 1A), will be used by another enzyme P5C reductase (P5CR) to produce proline.

**Figure 1—figure supplement 1.**
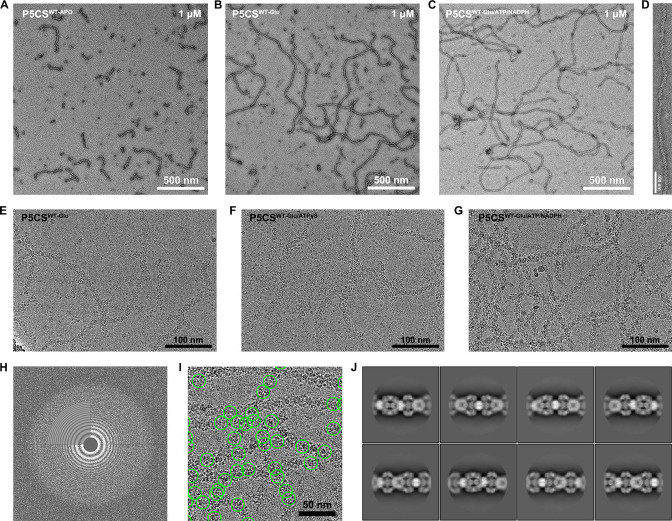
Substrates can significantly extend the P5CS filament. (**A**) Negative stain electron microscopy micrograph of P5CS protein in the APO state. P5CS protein in the APO state can self-assemble into filaments of various lengths. (**B**) When glutamate was added to the P5CS^APO^ protein, the extension of P5CS filament was observed compared to P5CS^APO^ filament. (**C**) When all substrates (MgSO_4_, ATP, NADPH, and glutamate) were added to induce the reaction, the long P5CS^Mix^ filaments were observed, which is similar to the P5CS^Glu^ filaments. (**D**) Representative negative stain electron microscopy micrographs of a single P5CS^Mix^ filament. (**E–G**) Representative cryo-EM micrograph of P5CS filaments in three ligand states. (**H**) The power spectrum of a micrograph showing simulated contrast transfer function rings. (**I**) The green circles represent single particles of the picked P5CS^Mix^ filaments. (**J**) Representative 2D class averages of the P5CS^Mix^ filament; several classes of P5CS filament particles with less curvature were selected.

**Figure 1—figure supplement 2.**
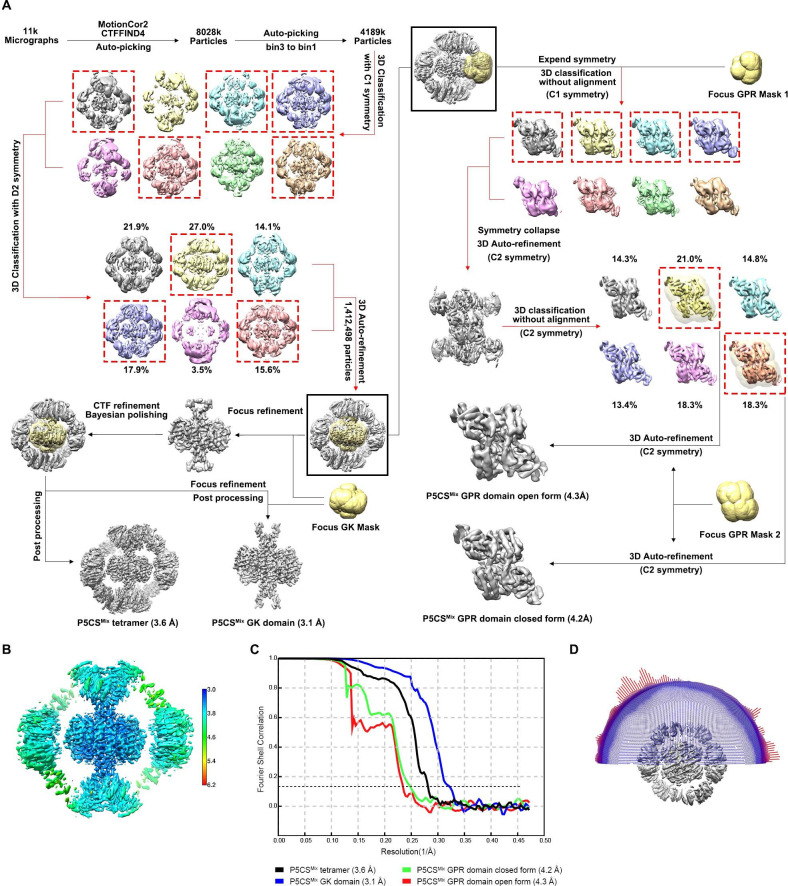
Cryo-electron microscopy (cryo-EM) analysis of the P5CS^Mix^ filament. (**A**) Flow chart for the cryo-EM reconstruction of the P5CS^Mix^ filament. The same processing scheme was used for the P5CS^Glu^ and P5CS^Glu/ATPγS^ filaments. Detailed procedures are described in Materials and methods. (**B**) Local resolution map (postprocessing) of the P5CS^Mix^ tetramer form of filament. (**C**) The gold-standard Fourier shell correlation (FSC) curves for four refined maps. The resolution calculated at FSC = 0.143 is indicated. (**D**) Angular distribution of the particles used for the final reconstruction of the P5CS^Mix^ filament.

**Figure 1—figure supplement 3.**
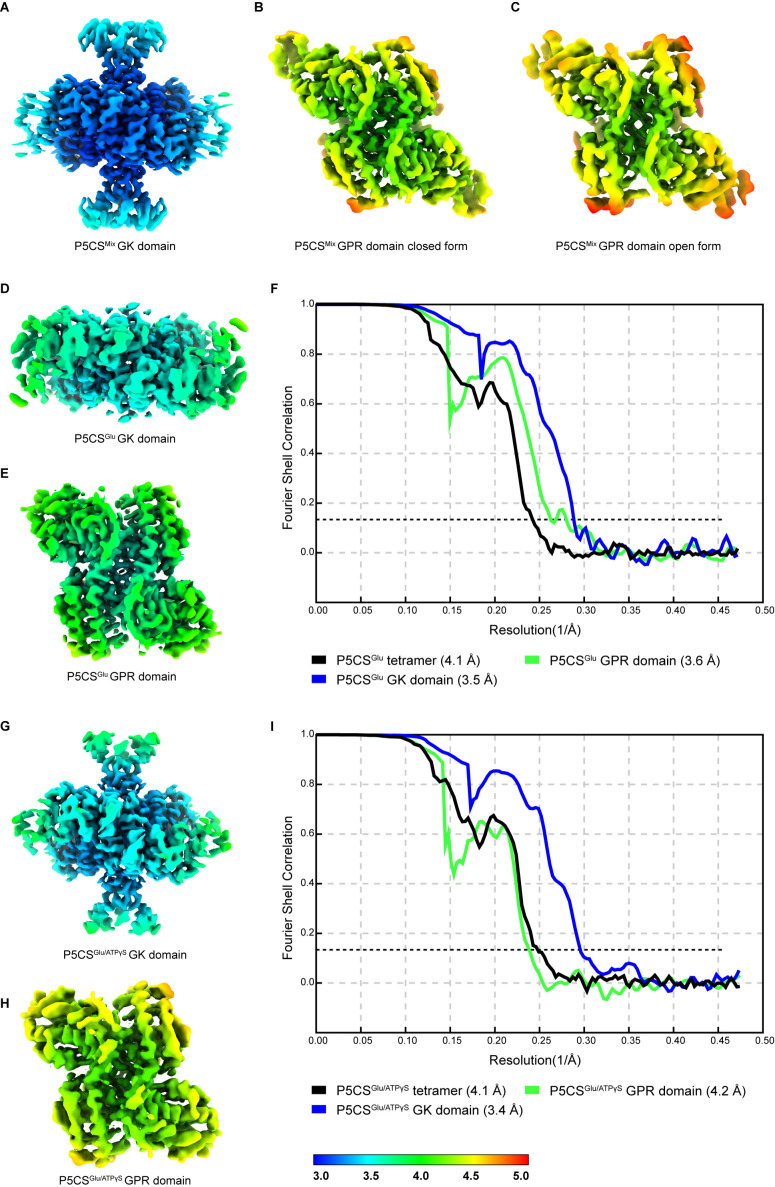
Quality of cryo-electron microscopy (cryo-EM) maps. (**A–C**) Local resolution distribution of the glutamate kinase (GK) and γ-glutamyl phosphate reductase (GPR) domains in the P5CS^Mix^ filament. The corresponding Fourier shell correlation (FSC) curve is shown in Figure 1—figure supplement 2. (**D–F**) Local resolution distribution of the GK and GPR domains in the P5CS^Glu^ filament, and FSC curve for all structures. The resolution calculated at FSC = 0.143 is indicated. (**G–I**) Local resolution distribution of the GK and GPR domains in the P5CS^Glu/ATPγS^ filament, and FSC curve for all structures. The resolution calculated at FSC = 0.143 is indicated.

**Figure 1—figure supplement 4.**
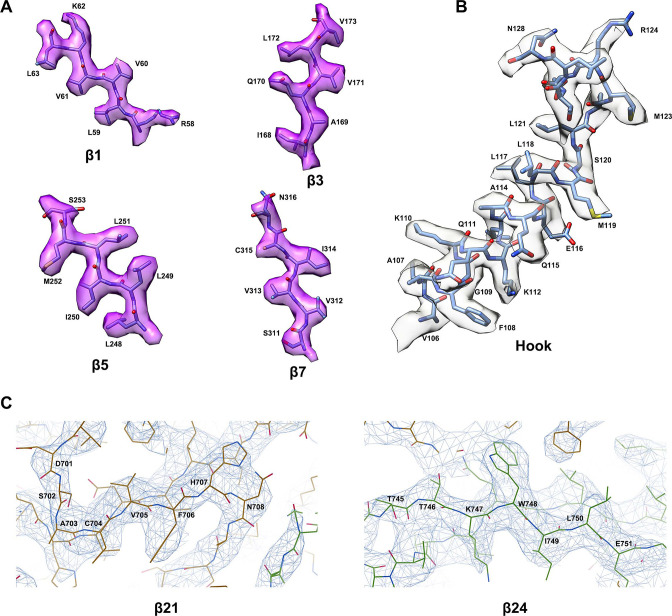
Representative cryo-electron microscopy (cryo-EM) map. Representative regions of the P5CS protein model superimposed by cryo-EM map. (**A**) Atomic model of P5CS β-strands. (**B**) Atomic model of the hook structure α-helices. (**C**) Representative cryo-EM density of β21 and β24 is displayed at 4.5 σ contour level; panel (**C**) was generated by Coot.

**Figure 1—video 1.** Morph between the consensus structures of P5CS^Glu^ filaments. The model was generated by fitting the tetramer into each class of the 3D classification with C1 symmetry. Protomers were colored differently. This video implies the dynamic changes of the Glu-bound state of P5CS filaments. Morphs between conformations were created in ChimeraX.

**Figure 1—video 2.** Morph between the consensus structures of P5CS^Mix^ filaments. The model was generated by fitting the tetramer into each class of the 3D classification with C1 symmetry. Each protomer was colored differently. This video shows the dynamic changes of P5CS^Mix^ filaments. Morphs between conformations were created in ChimeraX. See also Figure 1—video 1.

In order to solve the structure of P5CS filaments, we expressed and purified *Drosophila melanogaster* full-length P5CS proteins. First, we analyzed the APO and substrate-bound states of P5CS by negative staining (Figure 1—figure supplement 1A–D). In our previous study, we found that *Drosophila* P5CS in the APO state is hard to form filaments at low concentrations (<0.05 μM). The addition of glutamate to the P5CS samples induces micron-scale filaments (Zhang et al., 2020). Here, we observe that increasing P5CS concentration (>1 μM) also promotes the formation of filaments in the APO state. Our results show that the P5CS proteins can be self-assembled into filaments without ligands, and adding all substrates increases the length of filaments at the same concentration of the P5CS proteins. Consistent with our previous study, glutamate (a substrate of P5CS) promotes the formation and maintenance of *Drosophila* P5CS filaments (Zhang et al., 2020).

Subsequently, samples of the P5CS proteins incubated with different combinations of substrates were prepared for cryo-EM (Figure 1—figure supplement 1E–J). Filaments in three conditions with (1) glutamate (P5CS^Glu^), (2) glutamate and ATPγS (P5CS^Glu/ATPγS^), and (3) glutamate, ATP, and NADPH (P5CS^Mix^) were imaged in cryo-EM for single-particle analysis (SPA). Long and flexible filaments of P5CS were observed under all the three conditions. After 3D classification and 3D reconstruction, the electron density maps of the P5CS^Glu^, P5CS^Glu/ATPγS^, and P5CS^Mix^ filaments reached resolutions of 4.0 Å, 4.2 Å, and 3.6 Å, respectively (Figure 1B–D, Figure 1—figure supplements 2–4). Using a separate focused refinement strategy, we obtained multiple conformational states of the GK domain tetramer (3.1–3.5 Å) and the GPR domain dimer (3.6–4. 3Å). The cryo-EM data and model refinement statistics are provided in Table 1. The N-terminus (residues 1–44) and three disordered segments in regions I, II, and III in the GK domain were invisible in our maps.

**Table 1.** Cryo-electron microscopy (cryo-EM) data statistics.

	P5CS^Glu^ filament			P5CS^Glu/ATPγS^ filament			P5CS^Mix^ filament			
**Data collection and processing**										
EM equipment	Titan Krios			Titan Krios			Titan Krios			
Detector	K3 camera			K3 camera			K3 camera			
Magnification	22,500×			22,500×			22,500×			
Voltage (kV)	300			300			300			
Electron exposure (e–/Å^2^)	72			72			72			
Defocus range (μm)	–0.8 to –2.5			–0.8 to –2.5			–0.8 to –2.5			
Pixel size (Å)	0.53			0.53			0.53			
Symmetry imposed	D2			D2			D2			
Number of collected movies	4933			6408			10,566			
Initial particle images (no.)	1,911,843			1,563,553			8,027,582			
Final particle images (no.)	432,746			327,841			1,412,498			
**Refinement**										
	**P5CS tetramer**	**GK domain**	**GPR domain**	**P5CS tetramer**	**GK domain**	**GPR domain**	**P5CS tetramer**	**GK domain**	**GPR domain closed form**	**GPR domain open form**
EMDB ID	EMD-31466	EMD-31469	EMD-32877	EMD-31467	EMD-32876	EMD-32880	EMD-31468	EMD-32875	EMD-32878	EMD-32879
PDB code	7F5T	7F5X	7WXF	7F5U	7WX4	7WXI	7F5V	7WX3	7WXG	7WXH
Initial model used (PDB code)	-	4Q1T	2H5G	-	4Q1T	2H5G	-	4Q1T	2H5G	2H5G
Map resolution (Å)	4.1	3.5	3.6	4.1	3.4	4.2	3.6	3.1	4.2	4.3
FSC threshold	0.143	0.143	0.143	0.143	0.143	0.143	0.143	0.143	0.143	0.143
Map resolution range (Å)	3.8–8.0	3.4–5.2	3.5–5.0	3.4–8.0	3.2–4.7	4.1–5.3	3.3–7.8	3.0–4.1	4.1–5.9	4.0–5.5
Map sharpening B-factor (Å^2^)	–120	–120	–120	–100	–70	–200	–80	–80	–150	–150
*Model composition*										
Non-hydrogen atoms	20,436	7244	6,494	20,744	7968	6522	20,912	8172	6494	6590
Protein residues	2700	1896	860	2740	1040	860	2760	1064	860	430
Ligands	GGL	GGL	-	-	-	RGP	-	RGP, ADP	NAP	-
Ions	0	0	0	0	0	0	0	Mg	0	0
*B factors* (Å^2^)										
Protein	140	150	162	143	74	121	131	62	123	100
Ligand	140	150	-	-	-	145	-	-	-	121
*R.m.s. deviations*										
Bond lengths (Å)	0.005	0.005	0.007	0.007	0.006	0.007	0.005	0.006	0.008	0.005
Bond angles (°)	0.678	0.54	0.777	0.786	0.576	0.788	0.675	0.554	0.864	0.73
*Validation*										
MolProbity score	2.73	2.53	2.25	2.47	2.51	2.59	2.17	1.85	2.89	2.19
Clashscore	47.48	7.93	13.17	23.56	8.2	24.57	16.57	5.45	34.6	12.87
Poor rotamers (%)	0	6.12	0.29	0	6.39	0.58	0.18	3.56	1.74	0.29
*Ramachandran plot*										
Favored (%)	89.04	91.67	87.15	87.59	92.8	81.78	92.96	97.27	93.29	89.25
Allowed (%)	10.51	7.89	12.62	12.26	7.2	18.22	6.89	2.73	16.71	10.75
Disallowed (%)	0.45	0.44	0.23	0.15	0	0.58	0.15	0	0	0

GPR: γ-glutamyl phosphate reductase; GK: glutamate kinase; FSC: Fourier shell correlation.

One P5CS monomer can be roughly divided into five subdomains: (1) the glutamate-binding domain (GBD) and (2) the ATP-binding domain (ABD) at the GK domain; (3) the NADPH-binding domain (NBD), (4) the catalytic domain (CD), and (5) the oligomerization domain (OD) at the GPR domain (Figure 1A and E). In the model, two P5CS monomers dimerize through the interaction between their GPR domains, where the β21 at the CD interacts with the β24 at the OD of the other monomer (Figure 1F). This interaction connects two groups of hairpins and maintains the homodimer structure by a hydrogen bond network. Two P5CS dimers further assemble into a compact tetramer through the interaction at the GK domains. The P5CS tetramer serves as the building block of P5CS filaments (Figure 1G).

P5CS filament structures in all the three states showed characteristics of double helix (Figure 1—video 1, Figure 1—video 2). We chose the P5CS^Mix^ filament to display the details (Figure 1H). In the helical P5CS filament structure, the GK tetramers serve as the core of the filament, and the GPR dimers form left-handed double helix structures around the central axis. The overall diameter of P5CS filaments in all three states is 180 Å, while the helical twist is 68° and the helical rise is 60 Å (Figure 1H).

### Structural comparison of ligand-bound GK domains

The GK domain of *Drosophila* P5CS is conserved with the GK protein in *E. coli*. Alignments of sequences and structures indicate that their secondary structures are similar as both exhibit a sandwich-like α3β8α4 topological folding (Figure 2—figure supplement 1A), which is a characteristic of the amino acid kinase (AAK) family (Marco-Marín et al., 2007; Pérez-Arellano et al., 2010; Ramón-Maiques et al., 2002).

**Figure 2.**
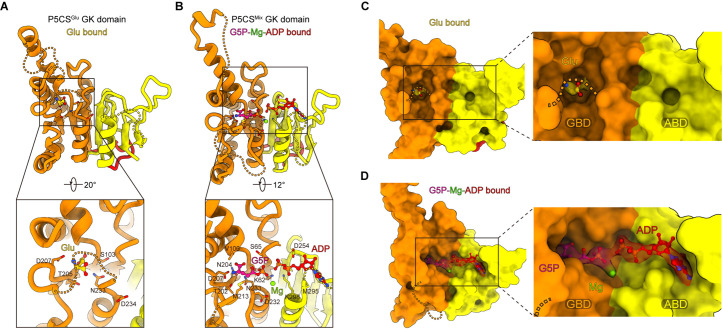
Conformational changes in the glutamate kinase (GK) domain-binding pocket. (**A**) GK domain of the P5CS^Glu^ filament, with glutamate shown as sticks with yellow carbons. The dashed lines represent disordered segments (residues 124–142, 211–232, and 275–297) in this model. (**B**) GK domain of the P5CS^Mix^ filament, with G5P, Mg^+^, and ADP shown as sticks with pink, green, and red carbons, respectively. The dashed lines represent disordered segments (residues 128–140, 214–228, and 282–295) in this model. (**C, D**) GK domain model surface representation showing the conformation of the binding pocket in the P5CS^Glu^ filament or P5CS^Mix^ filament. The cryo-electron microscopy (cryo-EM) density of binding glutamate molecule in (**C**), and the binding complex of G5P, Mg^+^, and ADP in (**D**). The dashed lines represent ‘open loop’ and ‘closed loop’

We obtained a structure of the GK domain with the binding of glutamate in the P5CS^Glu^ filament (Figure 2A) and a second structure of the GK domain with G5P-Mg-ADP in the P5CS^Mix^ filament (Figure 2B, Figure 2—figure supplement 1B). In the P5CS^Glu/ATPγS^ filament, the ligands could not be determined due to incomplete densities (Figure 2—figure supplement 1C). The GK domain structure of the P5CS^Glu/ATPγS^ filament is virtually identical to that of the P5CS^Mix^ filament (Figure 2—figure supplement 1D). We speculate that there are two ligand-binding modes (bound with Glu-Mg-ATPγS and G5P-Mg-ADP, respectively) in the P5CS^Glu/ATPγS^ filament. These two modes may coexist in the active sites of the GK tetramer, thereby affecting the 3D reconstruction of the structures. The unexpected presence of G5P could be due to the contamination of ATP in the commercial ATPγS (80% pure) and all substrates were in excess during our sample preparation. Thus, no ligand was modeled in the GK domain structure of the P5CS^Glu/ATPγS^ filament.

**Figure 2—figure supplement 1.**
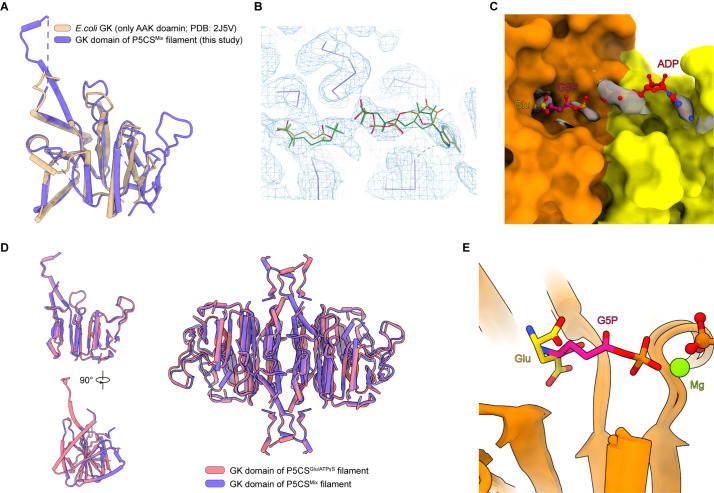
Structural details of the glutamate kinase (GK) domain characterized. (**A**) The comparison of *E. coli* GK structure without PUA domain (tan; PDB: 2J5V) and *Drosophila* GK domain structure of P5CS (blue-violet; this study), with 30.56% sequence identity and root-mean-square-deviation (RMSD) value of 1.363 Å (198 atom pairs). (**B**) Cryo-electron microscopy (cryo-EM) map quality of the GK domain active site in the P5CS^Mix^ filament (blue-violet), indicating that the conformation of bound G5P/ADP (green) is better than Glu/ATP (dark yellow). (**C**) Unmodeled ligand densities in the GK domain of the P5CS^Glu/ATPγS^ filament. (**D**) Superimposition between the GK domain protomer or tetramer structure of the P5CS^Glu/ATPγS^ filament (coral) and that of the P5CS^Mix^ filament (blue-violet). (**E**) The binding mode of glutamate (yellow) and G5P (pink) in the GK domain.

In the GK domain, a valley-like pocket locates between GBD and ABD, providing the binding sites for glutamate, ATP, or their derivatives (Figure 2C and D). Glutamate binds to the active site of GBD vertically (Figure 2A and C, Figure 2—figure supplement 1E). In contrast, G5P and ADP extend towards each other in the P5CS^Mix^ filament, and glutamate at the binding site is converted into the intermediate G5P. At ABD of the P5CS^Mix^ filament, the phosphate donor ATP becomes an ADP, associating with an Mg^2+^ (Figure 2B and D).

**Figure 3.**
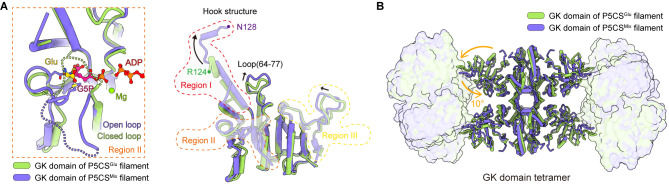
Structural comparison of the two types of glutamate kinase (GK) domain. (**A**) Comparison of one protomer of the GK domain tetramer in the P5CS^Glu^ filament (green) and P5CS^Mix^ filament (blue-violet) on the right panel. On the left panel, the dashed lines in the model represent the open loop (blue-violet) and closed loop (green) in region II. (**B**) Superimposition of the GK domain tetramer in the P5CS^glu^ filament (green) with the P5CS^Mix^ filament (blue-violet). Transitions from glutamate-bound-conformation to G5P-Mg-ADP-bound conformation are shown as curved arrows, indicating GK domain conformational changes in the P5CS filament.

Superimposing the GK tetramer in the P5CS^Glu^ filament and that in the P5CS^Mix^ filament revealed that the major motion of the GK domain occurred at the region containing flexible loops or disordered segments, whereas the α3β8α4 fold showed minor movement (Figure 3A). Meanwhile, based on the disorder densities in region II (Figure 3—figure supplement 1A–D), we modeled the possible trend of the missing segment with a dashed line (Figure 3A). In the P5CS^Glu^ filament, we speculate that the disordered segment in region II acts as a closed loop, which traps glutamate in GBD (Figures 2C and 3A). In the P5CS^Mix^ filament, the same segment shifts away from the top of the binding pocket and forms an open loop, in which residue M213 interacts with G5P (Figure 3A, Figure 3—figure supplement 1E). We notice that the closed loop has a steric clash with G5P, preventing the binding of G5P under such a conformation (Figure 3—figure supplement 1D). Our findings support the idea that region II at the GK domain engages in regulating the catalytic reaction.

**Figure 3—figure supplement 1.**
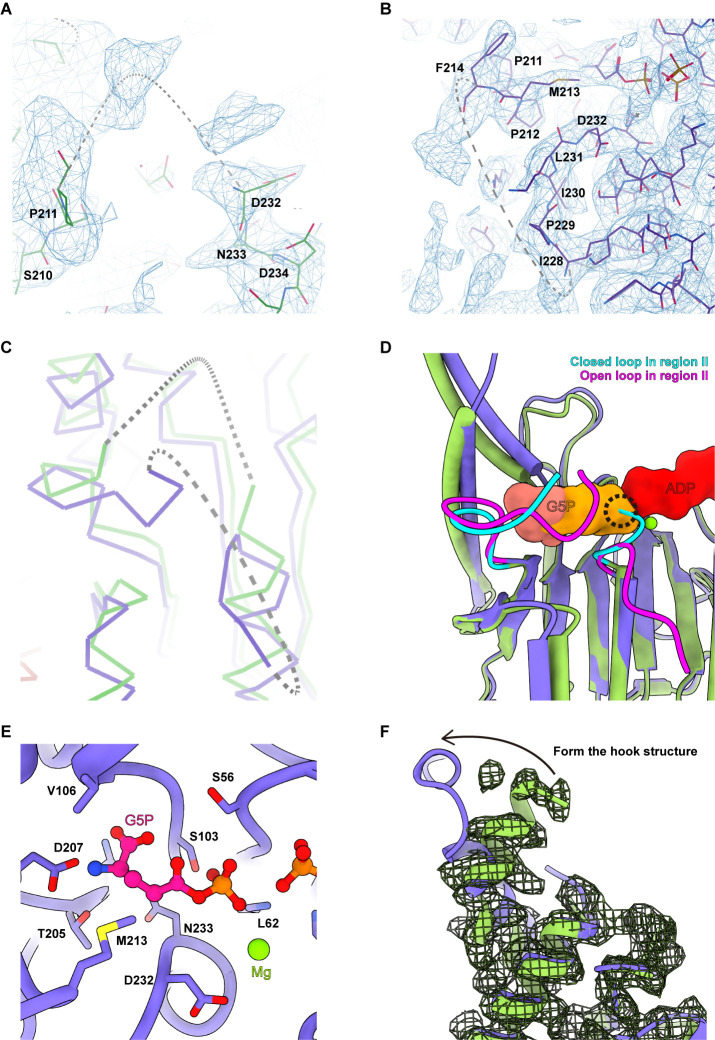
The conformational changes in regions I and II. (**A, B**) Cryo-electron microscopy (cryo-EM) map quality of region II in the P5CS^Glu^ filament (**A**) and P5CS^Mix^ filament (**B**). (**C**) Dashed lines generated by Coot represent disorder segment; superimposition between the P5CS^Glu^ filament (green) and P5CS^Mix^ filament (blue-violet) in region II indicates significant conformational differences. (**D**) The open loop (pink) and closed loop (cyan) are highlighted in our models, referred to panel (**C**). G5P (pink and yellow) and ADP (red) are shown as surface representation, the steric clash between the closed loop (cyan) and the phosphate moiety (yellow) of G5P is indicated. (**E**) G5P-binding site of the GK domain in the P5CS^Mix^ filament. (**F**) The conformational change of hook structure in region I, and cartoon models have overlaid the cryo-EM density of the P5CS^Glu^ filament shown as mesh.

The helix-helix structure (residues 105–113, 115–124) at region I of the P5CS^Glu^ filament transforms into a helix-loop-helix structure (residues 105–119, 120–122, 123–128) in the P5CS^Mix^ filament (Figure 3A, Figure 3—figure supplement 1F). This helix-loop-helix structure is referred to as the ‘hook’ structure. The transformation of the hook structure results in new contact sites between neighbor tetramers in the vertical direction, which is evidenced by a rigid density in our map (Figure 1B–D, Figure 1—figure supplements 2 and 3).

On the other hand, we notice the conformational variation of the loop at region III and a loop (residues 64–77) of GBD shifting greatly by approximately 3 Å away (Figure 3A). The function of these conformational changes is unclear, which may relate to conformational changes of the active site. In order to investigate the conformational changes involved in the catalytic reaction, we further compared the tetramer structures of the GK domain in the P5CS^Glu^ and P5CS^Mix^ filaments (Figure 3B). The GK domain of each protomer rotates approximately 10° around its central axis, causing the horizontal compression of the GK domain dimer. By comparing the structures of the GK domain with various ligands, we demonstrate the conformational changes, which may be associated with phosphorylation of the substrate glutamate.

### Open and closed conformations of GPR domains

The GPR domain of P5CS belongs to the aldehyde dehydrogenase (ALDH) superfamily. ALDH family uses NAD(P)^+^ to catalyze the conversion of various aldehydes into their corresponding carboxylic acids. Many studies on ALDHs have shown that a conserved residue cysteine acts as the active site of nucleophile, forming thiohemiacetal intermediate with substrate (Koppaka et al., 2012; Liu et al., 1997; Perozich et al., 1999). Curiously, the NADPH-utilizing GPR domain of P5CS catalyzes the reverse reaction of ALDHs.

**Figure 4.**
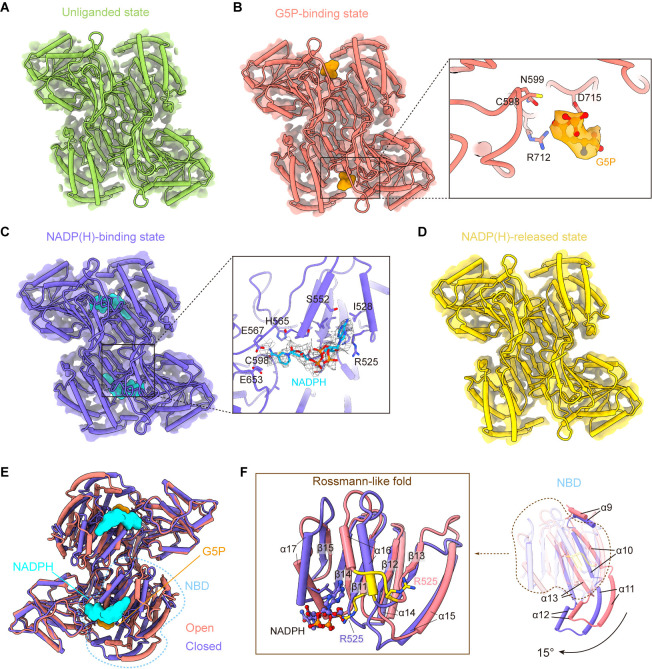
γ-Glutamyl phosphate reductase (GPR) domain ligand-bound mode and its conformation. (**A**) The cryo-electron microscopy (cryo-EM) density of the GPR dimer structure and cartoon model is represented as an unliganded state in the P5CS^Glu^ filament (green). (**B**) GPR dimer structure of the G5P-binding state in the P5CS^Glu/ATPγS^ filament (coral). The conformation of the G5P-binding pocket and G5P (orange) is shown as stick representation. (**C**) GPR dimer structure of the NADP(H)-binding state in the P5CS^Mix^ filament (blue-violet). The conformation of the NADP(H)-binding pocket with NADPH (cyan) is shown as stick representation. (**D**) GPR dimer structure of the NADP(H)-released state in the P5CS^Mix^ filament (yellow). (**E**) Structural differences in the G5P-binding state (coral) and NADP(H)-binding state (blue-violet) of the GPR domain. Ligands are colored as in (**B, C**). (**F**) Superimposition of either the NADPH-binding domain (NBD) or the Rossmann-fold of the GPR domain at the G5P-binding state and NADP(H)-binding state using a single protomer.

On the basis of P5CS structures, we display four different binding modes (Figure 4A–D) of the GPR domain. In the P5CS^Glu^ filament, no ligand binds to the GPR domain (Figure 4A). In the P5CS^Glu/ATPγS^, however, we observed the density of a G5P at the CD active site (Figure 4B, Figure 4—figure supplement 1A). It might be a contamination of ATP, leading to the production of the substrate G5P. In this model, the binding mode of G5P (referred to as the G5P-binding state) is clearly solved. By focus refinement of the GPR dimer in the P5CS^Mix^ filament, we determined two additional states of the GPR domain (Figure 4C and D). One is the NADP(H)-binding state, when NADP(H) is present at NBD (Figure 4—figure supplement 1B). The other is the NADP(H)-released state, of which the cofactor binding site is empty.

**Figure 4—figure supplement 1.**
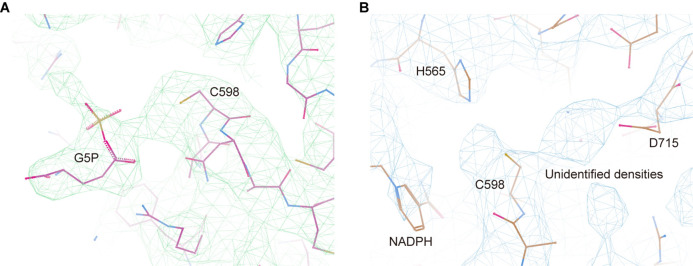
Representative cryo-electron microscopy (cryo-EM) densities for the active site of the γ-glutamyl phosphate reductase (GPR) domain. (**A**) Cryo-EM map quality of G5P ligand in the active site of the GPR domain in the P5CS^Glu/ATPγS^ filament. (**B**) Unmodeled densities in the active site of the GPR domain at the NADP(H)-binding state, which may be the reaction product: Pi or GSA/P5C.

**Figure 4—figure supplement 2.**
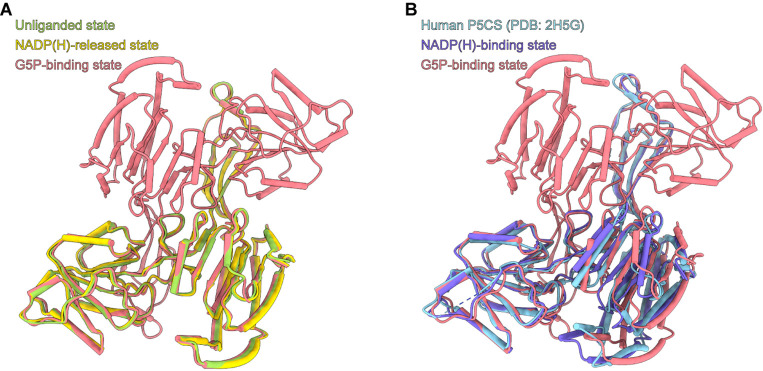
Comparison of the structures of the γ-glutamyl phosphate reductase (GPR) domain. (**A**) Superimposition of the GPR domain at the unliganded state (green), GPR domain at the NADP(H)-released state (yellow), and GPR domain dimer structure at the G5P-binding state (coral). (**B**) Superimposition of the GPR domain at the NADP(H)-binding state (blue-violet) and GPR domain dimer structure at the G5P-binding state (coral). Compared with the GPR domain of *Drosophila* P5CS at the NADP(H)-binding state (blue-violet), the GPR domain of human P5CS (cyan, PDB: 2H5G) has 56.74% sequence identity and root-mean-square-deviation (RMSD) value of 1.531 Å (398 atom pairs).

**Figure 4—figure supplement 3.**
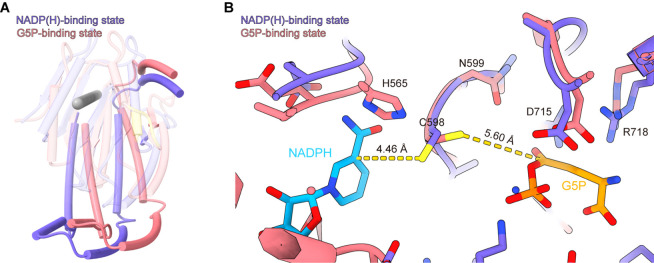
The NADPH-binding domain (NBD) rotation and view of the active site of the γ-glutamyl phosphate reductase (GPR) domain with its substrate. (**A**) The position of cylinder axes (gray) around which the NBD rotates. (**B**) Superimposition of the GPR domain at the G5P-binding state (coral) to that at the NADP(H)-binding state (blue-violet) on their active site; the distance between the ligands and catalytic residue C598 is shown by the dash line (yellow).

**Figure 4—video 1.** Structural transition of open and closed conformations of the γ-glutamyl phosphate reductase (GPR) domain. Morph from the G5P-binding state (open conformation) to the NADP(H)-binding state (closed conformation) of the GPR domain.

Conformational comparison of unliganded, G5P-binding and NADP(H)-released states shows that the overall structures of the GPR domain are similar (Figure 4—figure supplement 2A). The structure of the NADP(H)-release state, which has no bound ligand, is identical to the unliganded state. The binding of G5P to CD of the GPR domain does not lead to obvious conformational changes.

Next, we compared the structures of the G5P-binding state and NADPH-binding state (Figure 4E, Figure 4—figure supplement 2B). We have found that the structures of CD and OD are generally consistent in those two states, while NBD of the GPR domain in those two states differs greatly (Figure 4E). NBD contains consecutive alternating α-helices and β-strands (α_2_-β_5_-α_2_) architecture, which is known as the Rossmann-like fold for dinucleotide binding (Cheek et al., 2005). By superimposing the Rossmann-like fold and the entire NBD, we determined conformational changes between the GPR domain at the G5P-binding state and that at the NADP(H)-binding state (Figure 4F).

Upon NADP(H) binding, the residue R525 interacts with the adenine moiety. This interaction transforms the ^525^REE^527^ loop into an ordered structure that extends the α16 helix in the Rossmann-like fold (Figure 4F). Meanwhile, the entire NBD rotates approximately 15° along the cylinder axis (Figure 4—figure supplement 3A) and slides towards CD (Figure 4F; Figure 4—video 1). We hypothesize that the helix, when turns disordered, loses contact with the adenine moiety and then separates the cofactor from NBD via a conformational selection mechanism. A similar phenomenon was also observed in ALDH1L1 (Tsybovsky and Krupenko, 2011). This transformation contributes to bringing the nicotinamide ring of the NADP(H) close to the catalytic residue C598 of CD. Conformational changes triggered by the binding of NADPH subsequently initiate the transfer of the hydride ion from NADPH to the intermediate G5P (Figure 4—figure supplement 3B).

In our models, unliganded, G5P-binding and NADP(H)-released states represent as open conformation, and the NADP(H)-binding state represents as closed conformation. We propose that the P5CS filament accommodates the GPR domain in both open and closed conformations. Therefore, recurring transformations between these two conformations are essential for the catalytic cycle at the GPR domain.

### Filamentation regulates the enzymatic reaction

**Figure 5.**
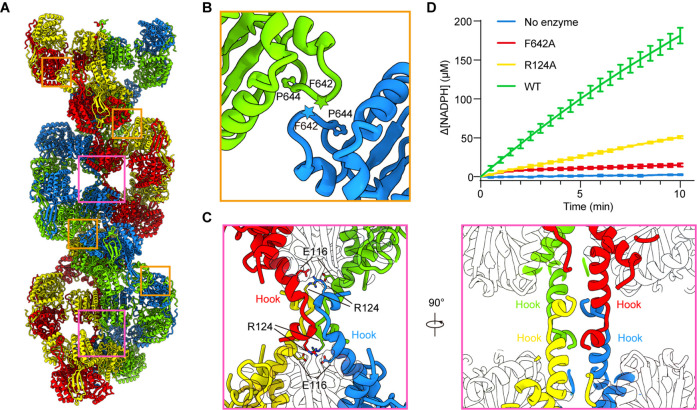
Assembly and interaction surfaces of the P5CS filament. (**A**) P5CS filament assembly interface, the four P5CS protomers in one layer are colored in red, yellow, blue, and green. (**B**) Interaction between two adjacent γ-glutamyl phosphate reductase (GPR) domain dimers, residues F642 located at loop that interacts with P644 from another neighboring GPR domain dimer. (**C**) Model for hook structure interaction. (**D**) Enzyme activity analysis to examine P5CS wild-type or mutant proteins. All of the experiments were replicated three times (n = 3, mean ± SD). Figure 5—source data 1.Enzymatic activity of wild-type and mutant *Drosophila* P5CS.

In P5CS^Glu^, P5CS^Glu/ATPγS^, and P5CS^Mix^ filaments, neighboring GPR dimers interact with each other and form a helical structure. The interaction formed between F642-P644 of the contact loops in adjacent GPR domains, which appears as a CH/Pi interaction (Zondlo, 2013), is critical for the filamentation (Figure 5A and B, Figure 5—figure supplement 1). In P5CS^Glu/ATPγS^ and P5CS^Mix^ filaments, the additional interface between hook structures pairs locks adjacent GK tetramers. The hook structure extrudes from the GK domain. In a GK tetramer, four hooks extrude toward two opposite directions to form a ‘spinning top’ arrangement. Therefore, two pairs of hooks in a GK tetramer interact with their counterparts in two adjacent GK tetramers (Figure 5A and C). The hook interaction forms strong contacts via hydrogen bonds (M119-R124, L121-M123) and salt bridges (E116-R124). Therefore, a combination of the GPR contact (for the double helix) and GK contact (for the axis) stabilizes the P5CS filament.

**Figure 5—figure supplement 1.**
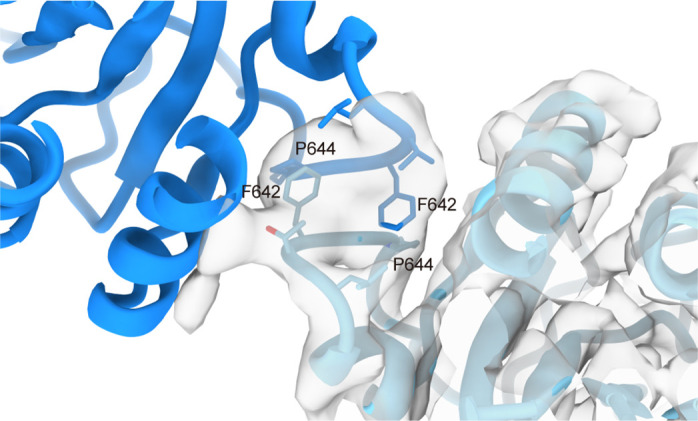
The interface of adjacent γ-glutamyl phosphate reductase (GPR) domain dimers. Close-up of the interaction interface, the key residues are shown as sticks, with the overlaid cryo-electron microscopy (cryo-EM) density of the glutamate kinase (GK) domain at unliganded state.

**Figure 5—figure supplement 2.**
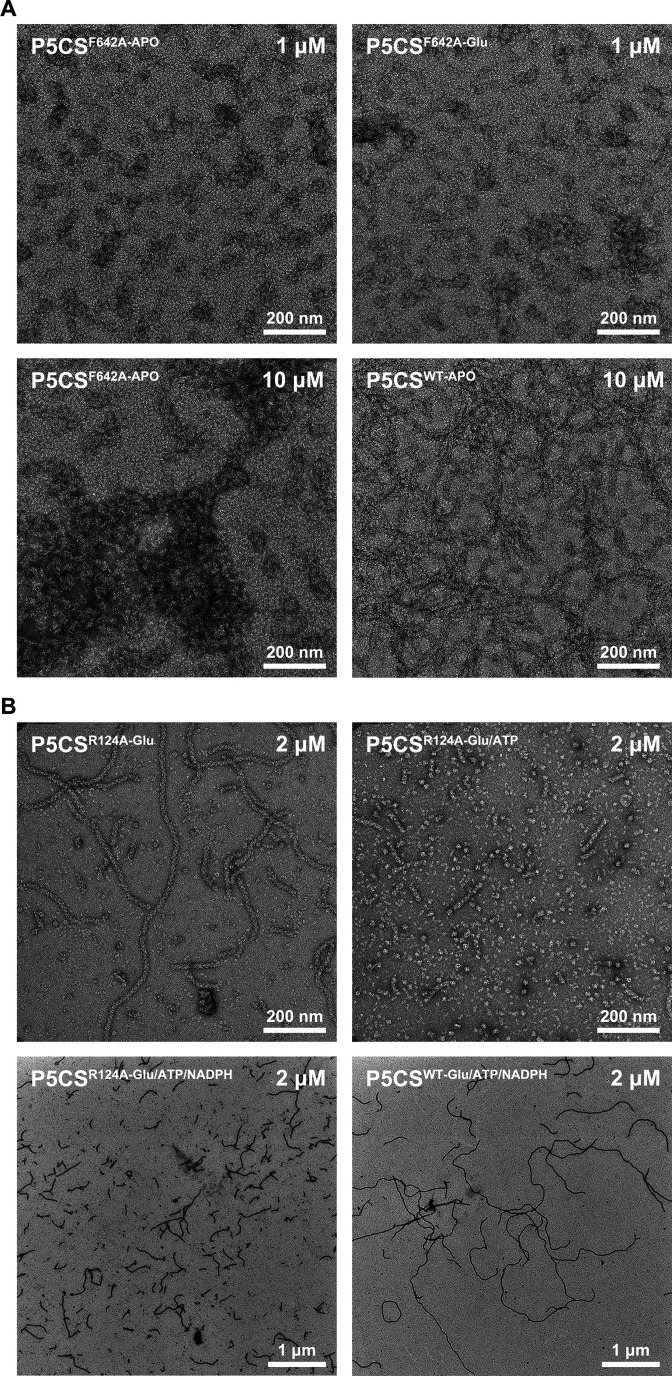
Negative staining of mutated P5CS. (**A**) Negative stain electron microscopy micrographs of P5CS^F642A^ mutation protein at the APO state; this mutation disrupts the filamentation of P5CS. (**B**) When the P5CS^R124A-Glu^ filament was additionally incubated with ATP, depolymerization of P5CS^R124A^ filament was observed.

**Figure 5—figure supplement 3.**
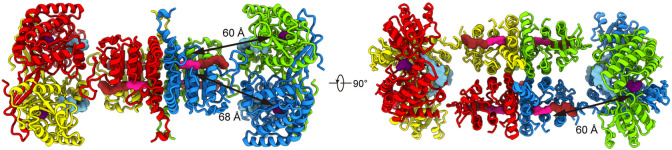
The distance between the active sites of the glutamate kinase (GK) domain and γ-glutamyl phosphate reductase (GPR) domain. The tetramer form of P5CS in filament; positions of each ligand are simulated in our models. G5P (pink) and ADP (brown) in the GK domain, G5P (purple), and NADPH (cyan) in the GPR domain are shown as surface representation. Distances between the G5P in the GK domain and GPR domain are indicated.

**Figure 5—figure supplement 4.**
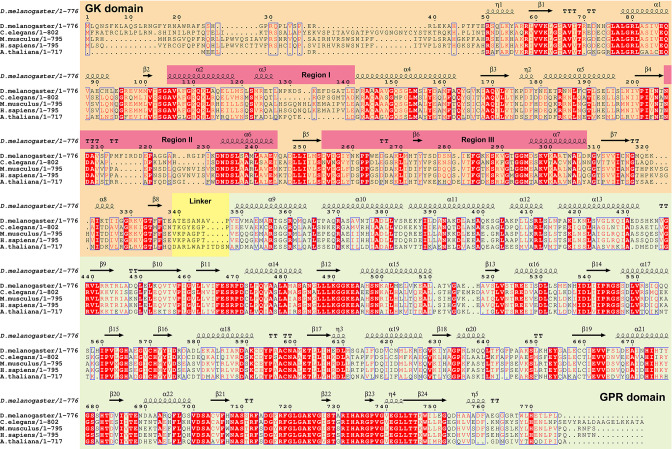
Sequence alignment of the representative P5CS enzymes. The sequence alignment of P5CS sequences of *Drosophila* (UniProtKB: Q9VNW6), mouse (UniProtKB: Q9Z110; isoform long), human (UniProtKB: P54886; isoform long), *C. elegans* (UniProtKB: P54889; isoform b), and *Arabidopsis* (UniProtKB: P54887; isoform 1). The conserved residues are identically shaded red, and secondary structure elements are indicated above.

**Figure 5—video 1.** Simulated ligand-binding site of P5CS filament. The structural models and color codes refer to Figure 5 and Figure 5—figure supplement 3.

To understand the function of P5CS filaments, we generated two mutants, R124A and F642A, which are predicted to abrogate the tetramer-tetramer contact sites of the GK domains and GPR domains, respectively. Negative stain of the mutant P5CS showed that the P5CS^F642A^ mutant proteins did not assemble into a filament with or without ligands (Figure 5—figure supplement 2A). These results indicate that the interaction at the GPR domain interface is crucial for P5CS filamentation.

In contrast, the P5CS^R124A^ mutant proteins formed long filaments in the APO state as well as in the presence glutamate (Figure 5—figure supplement 2B). We observed that glutamate-bound P5CS^R124A^ filaments disassembled at the initial phase of adding ATP. Being incubated with all substrates, P5CS^R124A^ formed shorter filaments than P5CS^WT^ (Figure 5—figure supplement 2B).

We propose that the interactions among the hook pairs are required for stabilizing the filament during the transformation from the P5CS^Glu^ filament to the P5CS^Glu/ ATPγS^ filament or P5CS^Mix^ filament. We subsequently analyzed the activity of the wild-type P5CS and two mutants, R124A and F642A. The two mutants exhibited a dramatically compromised activity in comparison with the wild-type P5CS (Figure 5D), suggesting that the integrity of filament is essential to the catalytic reactions.

## Discussion

### The GK domain

We observed two ligand-binding modes in the GK domain. Due to the lack of ATP-bound structure, it is difficult to determine whether ATP plays a decisive role in these conformational changes. According to a previous report on the N-acetyl-l-glutamate kinase (NAGK), nucleoside is important for the conformational change of the AAK domain, and the structures are similar when bound by ADP or AMPPNP (Gil-Ortiz et al., 2011). Based on the similarity of sequences and structures between GK and NAGK (Marco-Marín et al., 2007), we propose that the conformation of the GK domain in the P5CS^Glu^ filament would transform upon the binding of ATP, thereby triggering the formation of hook structure and completing the catalytic reaction. Although we solved the clear structure of the P5CS^Glu^ filament, further research is needed to understand how the conformation of glutamate binding contributes to the extension of P5CS filaments.

### The GPR domain

Aspartate-β-semialdehyde dehydrogenase (ASADH) catalyzes NADPH-dependent reductive dephosphorylation of β-aspartyl phosphate to aspartate-β-semialdehyde (Karsten and Viola, 1991). The GPR domain of P5CS and ASADH catalyzes the same type of reaction. Interestingly, the binding of NADP^+^ will change the cofactor binding domain of ASADH from open conformation to closed conformation (Hadfield et al., 2001). Thus, we speculate that their catalytic mechanisms have something in common. In the GPR domain of *Drosophila* P5CS, our data suggest that the catalytic residue C598 of CD attacks the G5P to form the first tetrahedral thioacetal intermediate in the reaction, and then expulsion of phosphate collapses to form a stable thioacyl enzyme intermediate. A hydride is then transferred to this intermediate from NADPH, with subsequent collapse to release the product GSA. Furthermore, the NADPH-binding site is located inside the filament, close to the GK domain. The G5P binding site is close to the external solution environment, which is proposed to facilitate the release of the product GSA/P5C (Figure 5—figure supplement 3, Figure 5—video 1). G5P can freely bind to the GPR domain in our model (G5P-binding state). However, in the closed conformation, when the nicotinamide ring of NADP(H) approaches the G5P-binding site, the substrate tunnel entrance may be blocked by NADP(H). This may affect the subsequent binding of G5P. Therefore, we speculate that the GPR domain should bind with G5P prior to NADPH binding. However, whether this mechanism is a preferred binding order needs to be further verified by kinetic experiments.

### The P5CS filament

As mentioned in the ‘Results’ section, we observed that mutated residues R124A and F642A do not directly participate in the active sites, while they are crucial for filamentation. This suggests that the P5CS filamentation couples the reaction catalyzed between the GK domain and GPR domain through transferring unstable intermediate G5P (Pérez-Arellano et al., 2010; Seddon et al., 1989). Considering the distance between the GK and GPR domains is about 60 Å (Figure 5—figure supplement 3, Figure 5—video 1), we propose a model that P5CS filament may exhibit a scaffold architecture that stabilizes the relative position of the GK and GPR domains, the cooperation between which may produce electrostatic substrate channels that mediate the transfer of unstable intermediate G5P. In addition, P5CS filamentation may create a half-opened chamber with the active sites located at the inner part of the filament. Since the GK domain is catalytically faster than the GPR domain, the unstable intermediates G5P accumulate within the filament. This microenvironment may reduce the amount of G5P escaped into the solvent, thereby facilitating the rate-limiting reaction at the GPR domain.

### The working model

**Figure 6.**
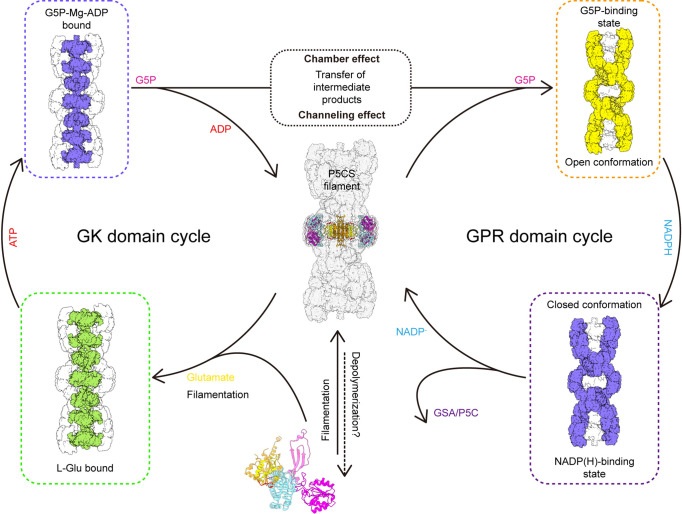
Model of P5CS filament structural transitions during GSA/P5C synthesis. The P5CS molecule polymerizes into filaments at the APO state or after binding with the glutamate. Upon ATP binding, the glutamate kinase (GK) domain initiates glutamate phosphorylation. The product leaves the pocket, and the GK domain subsequently repeats reaction cycle (left). Unstable G5P will be transported through channel and the half-open chamber inside the filament, and captured by the γ-glutamyl phosphate reductase (GPR) domain. NADPH binding to the GPR domain transforms the domain to closed conformation, which enables NADPH to approach the catalytic site and completes reductive dephosphorylation of G5P. The GSA/P5C will be released, and the GPR domain returns to the unliganded state with open conformation. The GPR domain then begins the next cycle (right).

Together, we propose a coupling catalytic reaction mechanism of *Drosophila* dynamic P5CS filament. In this proposed model, spontaneous filamentation occurs at the APO state, and elongation of P5CS filament is associated with the binding of glutamate. Upon the binding of glutamate, the binding pocket at the GK domain is bound by ATP; subsequently, conformational changes facilitate the formation of a hook structure and phosphorylation of glutamate, which produces G5P. When products of the GK domain dissociate from the active site, G5P would be trapped by the channel and chamber within the filament and further captured by the GPR domain. Next, NADPH binds to the GPR domain, triggering the conformational change into closed conformation, which brings the NADPH towards the catalytic residue C598 and facilitates the reaction. After this reaction, NADP^+^ and GSA will be released, and the GPR domain returns to its open conformation (Figure 6). This working model suggests that the GK and GPR domains undergo continuous conformational transition during catalysis, resulting in a dynamic filament.

In the P5CS^Glu^ filament, the GK and GPR domains are likely in a stable conformational state, while vibration may occur in the GPR domain of P5CS^Glu/ATPγS^ and P5CS^Mix^ filaments due to the binding of ligands. This notion could be supported by the differences in their local resolution (Figure 1B-D). We speculate that the swing of GPR in the catalytic reaction could destabilize the interaction between adjacent GPR domain dimers in the filament. Therefore, the extra interaction at the hook structure of the GK domain may be required for the stabilization of the filament. This proposed stabilization is consistent with negative stain data showing that the P5CS^R124A^ mutant cannot stabilize the filament structure in the catalytic process and lose the ability to form the long filaments.

### P5CS and human disease

Recently, accumulative evidences have shown that mutations on the human P5CS gene (*ALDH18A1*) is one of the causes of hyperammonemia, neurocutaneous syndromes, and motor neuron syndrome (Baumgartner et al., 2005; Magini et al., 2019; Marco-Marín et al., 2020; Pérez-Arellano et al., 2010). Such mutations may result in the loss of P5CS function in various degrees. *Drosophila* P5CS residue R124 in region I of the GK domain, which corresponds to R138 of human P5CS, is highly conserved among different eukaryotes (Figure 5—figure supplement 4). However, 17 residues in region I, including the hook structure, are absent in *E. coli* GK. Pathogenic mutations of R138 in human P5CS, which are proven to be the cause of autosomal-dominant cutis laxa, have been demonstrated with a decreased activity and a dispersed distribution in mitochondria (Fischer-Zirnsak et al., 2015; Yang et al., 2021). In the protein structure database, there is only the GPR domain structure available for human P5CS (PDB: 2H5G). Its overall structure is similar to the GPR domain of *Drosophila* P5CS (Figure 4—figure supplement 2B). Although it is still unknown whether human P5CS can form filament structure in vitro, it is reasonable to suspect that the filament-forming property is conserved between human and *Drosophila* P5CS based on their structural similarity. Our structure reveals that the R138 mutation on human P5CS could abrogate the interaction between hook structures of GK domains, and thereby destabilize the filament and coupling of reactions at the two domains.

In summary, our cryo-EM structures of *Drosophila* P5CS filament present the assembly mode of P5CS protein and provide a molecular basis for a further understanding of the reaction mechanism of the GK and GPR domains. In our proposed model, the coupling of the GK and GPR domains in the filamentous structure facilitates the catalytic reaction of the bifunctional enzyme P5CS. Additional structural studies of P5CS filaments are required to determine whether there is an underlying regulatory mechanism that transmits information between the GK and GPR domains in the tetramer and along the filament.

## Materials and methods

**Key resources table:** 

Reagent type (species) or resource	Designation	Source or reference	Identifiers	Additional information
Gene (*Drosophila melanogaster*)	P5CS	GenBank	NM_001259948	
Strain, strain background (*Escherichia coli*)	Transetta (DE3)	TransGen Biotech		
Recombinant DNA reagent	pET28a-6His-SUMO	In house		
Commercial assay or kit	BCA Protein Concentration Determination Kit (Enhanced)	Beyotime	P0010	
Chemical compound, drug	Benzamidine hydrochloride	Sigma-Aldrich	434760-5G	
Chemical compound, drug	Pepstatin A	Sigma-Aldrich	P5318-25MG	
Chemical compound, drug	Leupeptin hydrochloride microbial	Sigma/Aldrich	L9783-100MG	
Chemical compound, drug	PMSF	MDBio	P006-5g	
Chemical compound, drug	Ni-NTA Agarose	QIAGEN	30250	
Chemical compound, drug	l-Glutamic acid	Sigma-Aldrich	G1251-100G	
Chemical compound, drug	ATP	Takara	4041	
Chemical compound, drug	ATP-gamma-S	Abcam	ab138911	
Chemical compound, drug	NADPH tetrasodium salt	Roche	10107824001	
Other	Nitinol mesh	Zhenjiang Lehua Electronic Technology	M024-Au300-R12/13	Cryo-EM grid preparation
Other	Holey Carbon Film	Quantifoil	R1.2/1.3, 300 Mesh, Cu	Cryo-EM grid preparation
Other	400 mesh reinforced carbon support film	EMCN	BZ31024a	Negative staining
Software, algorithm	UCSF Chimera	https://doi.org/10.1002/jcc.20084		https://www.cgl.ucsf.edu/chimera
Software, algorithm	UCSF ChimeraX	https://doi.org/10.1002/pro.3235		https://www.cgl.ucsf.edu/chimerax/
Software, algorithm	RELION	https://doi.org/10.7554/eLife.42166		https://relion.readthedocs.io/en/latest/index.html#
Software, algorithm	Coot	https://doi.org/10.1107/S0907444910007493		https://www2.mrc-lmb.cam.ac.uk/personal/pemsley/coot/
Software, algorithm	Phenix	https://doi.org/10.1107/S2059798318006551		https://phenix-online.org/

### P5CS protein purification

The full-length *D. melanogaster* P5CS gene was cloned into a modified pET28a vector with a 6 × His SUMO tag fused at the N terminus; the fusion proteins were expressed in *E. coli* Transetta (DE3) cells overnight at 16°C after induction with 0.1 mM IPTG at OD_600_ range of 0.6–0.8. The remainder of purification was performed at 4°C. The harvested cells were sonicated under ice and purified by Ni-NTA agarose beads (QIAGEN) in lysis buffer (50 mM Tris-HCl pH 8.0, 500 mM NaCl, 10% glycerol, 20 mM imidazole, 1 mM PMSF, 5 mM β-mercaptoethanol, 5 mM benzamidine, 2 μg/ml leupeptin, and 2 μg/ml pepstatin). After in-column washing with lysis buffer, the proteins were eluted with elution buffer (50 mM Tris-HCl pH 8.0, 500 mM NaCl, 250 mM imidazole, 5 mM β-mercaptoethanol), peak fractions were treated with SUMO protease for 1 hr at 8°C. The P5CS proteins were further purified through HiLoad 16/600 Superdex 200pg gel-filtration chromatography (GE Healthcare) in column buffer (25 mM HEPES pH 7.5 and 100 mM KCl), peak fractions were collected, concentrated, and stored at –80°C before use.

### Enzyme activity assays

The full-length wild-type or mutant P5CS (100 nM protein) activity was determined in the reaction buffer containing 25  mM HEPES pH 7.5, and 10 mM l-glutamate (Sigma), with added 20  mM MgCl_2_, 10  mM ATP (Takara), and 0.5  mM NADPH (Roche) used to initiate the reaction (Magini et al., 2019; Sabbioni et al., 2021), then the reaction was monitored at 37°C in an MD-SpectraMax i3 plate reader and absorbance at 340 nm was measured every 20 s for 10  min (one experiment, n  =  3). The NADPH concentration was converted from A340 with the standard curve determined at the same experiment.

### Negative staining

Wild-type or mutation P5CS proteins were mixed with different substrate conditions. In brief, the final concentration was as follows: 25 mM HEPES pH 7.5, 100 mM KCl, 10 mM MgSO_4_, 100 mM l-glutamate, 10 mM ATP, and 0.5 mM NADPH. The prepared protein samples were applied to glow-discharged carbon-coated EM grids (400 mech, EMCN), and stained with 1% uranyl acetate. Negative-stain EM grids were photographed on a Tecnai Spirit G21 microscope (FEI).

### Cryo-EM grid preparation and data collection

For cryo-EM, purified full-length P5CS was diluted to approximately 2 μM and dissolved in buffer containing 25 mM HEPES pH 7.5, 100 mM KCl, 10 mM MgSO_4_, and incubated with 20 mM l-glutamate for the P5CS^Glu^ filament preparation. The P5CS^Glu/ATPγS^ filament was added with an additional 0.5 mM ATPγS (Abcam) compared to the P5CS^Glu^ filament. For the P5CS^Mix^ filament, P5CS proteins (2 μM) were incubated with 100 mM KCl, 10 mM MgSO_4_, 20 mM l-glutamate, 2 mM ATP, and 0.5 mM NADPH. All the samples were incubated for 1 hr on ice before vitrification. The P5CS filament samples were placed on H_2_/O_2_ glow-discharged holey carbon grids (Quantifoil Cu 300 mesh, R1.2/1.3) or amorphous alloy film (CryoMatrix M024-Au300-R12/13). Then, the grids were immediately blotted for 3.0 s and plunge-frozen in liquid ethane cooled by liquid nitrogen using Vitrobot (Thermo Fisher) at 4°C with 100% humidity. Images were collected on Titan Krios G3 (FEI) equipped with a K3 Summit direct electron detector (Gatan), operating in counting super-resolution mode at 300 kV with a total dose of 72 e^−^/Å^2^, subdivided into 50 frames in 4 s exposure using SerialEM (Mastronarde, 2005). The images were recorded at a nominal magnification of 22,500 × and a calibrated pixel size of 1.06 Å, with defocus ranging from 0.8 to 2.5 μm.

### Image processing and 3D reconstruction

The whole-image analysis was performed with RELION3 (Zivanov et al., 2018). We used MotionCor2 (Zheng et al., 2017) and CTFFIND4 (Rohou and Grigorieff, 2015) via RELION GUI to pr-process the image, movie frames were aligned, and the contrast transfer function (CTF) parameters were estimated in this process. After manual selection, there are 4933 images for the P5CS^Glu^ dataset, 6408 images for the P5CS^Glu/ATPγS^ dataset, and 10,566 images for the P5CS^Mix^ dataset left for further processing. For the flexibility of P5CS filaments, SPA was carried out in our reconstructions and no helical symmetry was implied in the whole process. Reference-free particle picking built in RELION3 was performed. This process provides 1,994,786 particles for P5CS^Glu^, 2,024,372 particles for P5CS^Glu/ATPγS^, and 8,027,582 particles for P5CS^Mix^. At first, the particles were extracted binning two or three times for the fast 2D classifications. Datasets were cleaned with several rounds of 2D classification and the bin factors were gradually reduced to one at the same time. After 3D classifications with C1 symmetry were applied, several classes were selected to do finer 3D classifications with D2 symmetry. Classes with the intact structure were retained for 3D refinement with D2 symmetry. For the 3D refinement, 432,746, 327,841, and 1,412,498 particles were used for each dataset. The maps including three P5CS tetramer layers were obtained. The relative motion between GK and GPR limited the refinement at a high resolution, so we used the partition reconstruction strategy to improve the resolution for both the GK and GPR domains. For the GK domain, we used continued local refinement to improve the resolution with a mask focus on the middle layer GK. Then, the Ctf-refinement and Bayesian polishing were performed for the remained particles and improved the resolution to 4. 1Å, 4.1 Å, and 3.6 Å for three-layer P5CS maps and 3.5 Å, 3.4 Å, and 3.1 Å for GK maps. For the GPR domain, particles were expended symmetry for the 3D classification without alignment. Several classes with the intact structure were selected and oriented; symmetry collapse was done at the same time. Then, 3D classifications and refinements with C2 symmetry were performed. For the P5CS^Mix^, two different states of GPR were captured. Finally, we got 286,291, 348,804, 193,482, and 233,624 particles to construct maps for the GPR domain with 3.6 Å, 4.2 Å, 4.3 Å, and 4.2 Å resolutions. LocalRes was used to estimate the local resolution of our map.

### Model building refinement and validation

Based on our maps with the near-atomic resolution, the model of the GK and GPR domains was generated with focused refinement maps in different states. The initial model of the GK and GPR domains was generated via swiss model regarding 4Q1T (GK from *Burkholderia thailandensis*) and 2H5G (human GPR domain) as a reference, respectively. Manual adjustment and building the missing regions were done in Coot (Emsley and Cowtan, 2004). Real space refinements were performed with Phenix (Adams et al., 2011). The full-length P5CS models were linked using the corresponding GK and GPR structures; the linker was generated in the Coot and refined via Phenix. Figures and movies were generated with UCSF Chimera (Pettersen et al., 2004) and ChimeraX (Goddard et al., 2018).

## Data Availability

Atomic models generated in this study have been deposited at the PDB under the accession codes 7F5T, 7F5U, 7F5V, 7F5X, 7WX3, 7WX4, 7WXF, 7WXG, 7WXH, 7WXI. Cryo-EM maps deposited to EMDB as: EMD-31466, EMD-31467, EMD-31468, EMD-31469, EMD-32875, EMD-32876, EMD-32877, EMD-32878, EMD-32879, EMD-32880. Source Data files have been provided for Figure 5D. The following datasets were generated: ZhongJ GuoCJ ZhouX LiuJL 2021*Drosophila* P5CS filament with glutamate.RCSB Protein Data Bank7F5T ZhongJ GuoCJ ZhouX LiuJL 2021GK domain of *Drosophila* P5CS filament with glutamateRCSB Protein Data Bank7F5X ZhongJ GuoCJ ZhouX LiuJL 2022GPR domain of *Drosophila* P5CS filament with glutamateElectron Microscopy Data BankEMD-32877 ZhongJ GuoCJ ZhouX LiuJL 2022GPR domain of *Drosophila* P5CS filament with glutamateRCSB Protein Data Bank7WXF ZhongJ GuoCJ ZhouX LiuJL 2021*Drosophila* P5CS filament with glutamate and ATPγSRCSB Protein Data Bank7F5U ZhongJ GuoCJ ZhouX LiuJL 2022GK domain of *Drosophila* P5CS filament with glutamate and ATPγSRCSB Protein Data Bank7WX4 ZhongJ GuoCJ ZhouX LiuJL 2022GPR domain of *Drosophila* P5CS filament with glutamate and ATPγSRCSB Protein Data Bank7WXI ZhongJ GuoCJ ZhouX LiuJL 2021*Drosophila* P5CS filament with glutamate, ATP, and NADPHRCSB Protein Data Bank7F5V ZhongJ GuoCJ ZhouX LiuJL 2022GK domain of *Drosophila* P5CS filament with glutamate, ATP, and NADPHRCSB Protein Data Bank7WX3 ZhongJ GuoCJ ZhouX LiuJL 2022GPR domain closed form of *Drosophila* P5CS filament with glutamate, ATP, and NADPHRCSB Protein Data Bank7WXG ZhongJ GuoCJ ZhouX LiuJL 2022GPR domain open form of *Drosophila* P5CS filament with glutamate, ATP, and NADPHRCSB Protein Data Bank7WXH ZhongJ GuoCJ ZhouX LiuJL 2021*Drosophila* P5CS filament with glutamateElectron Microscopy Data BankEMD-31466 ZhongJ GuoCJ ZhouX LiuJL 2021GK domain of *Drosophila* P5CS filament with glutamateElectron Microscopy Data BankEMD-31469 ZhongJ GuoCJ ZhouX LiuJL 2021*Drosophila* P5CS filament with glutamate and ATPγSElectron Microscopy Data BankEMD-31467 ZhongJ GuoCJ ZhouX LiuJL 2022GK domain of *Drosophila* P5CS filament with glutamate and ATPγSElectron Microscopy Data BankEMD-32876 ZhongJ GuoCJ ZhouX LiuJL 2022GPR domain of *Drosophila* P5CS filament with glutamate and ATPγSElectron Microscopy Data BankEMD-32880 ZhongJ GuoCJ ZhouX LiuJL 2021*Drosophila* P5CS filament with glutamate, ATP, and NADPHElectron Microscopy Data BankEMD-31468 ZhongJ GuoCJ ZhouX LiuJL 2022GK domain of *Drosophila* P5CS filament with glutamate, ATP, and NADPHElectron Microscopy Data BankEMD-32875 ZhongJ GuoCJ ZhouX LiuJL 2022GPR domain closed form of *Drosophila* P5CS filament with glutamate, ATP, and NADPHElectron Microscopy Data BankEMD-32878 ZhongJ GuoCJ ZhouX LiuJL 2022GPR domain open form of *Drosophila* P5CS filament with glutamate, ATP, and NADPHElectron Microscopy Data BankEMD-32879

## Additional files

Transparent reporting form
